# MR elastography in nonalcoholic fatty liver disease: inter-center and inter-analysis-method measurement reproducibility and accuracy at 3T

**DOI:** 10.1007/s00330-021-08381-z

**Published:** 2021-12-20

**Authors:** An Tang, Bogdan Dzyubak, Meng Yin, Alexandra Schlein, Walter C. Henderson, Jonathan C. Hooker, Timoteo I. Delgado, Michael S. Middleton, Lin Zheng, Tanya Wolfson, Anthony Gamst, Rohit Loomba, Richard L. Ehman, Claude B. Sirlin

**Affiliations:** 1 Department of Radiology, Radiation Oncology and Nuclear Medicine, Université de Montréal, Montréal, Québec, Canada; 2 Department of Radiology, Mayo Clinic, Rochester, MN USA; 3 Liver Imaging Group, Department of Radiology, University of California San Diego, San Diego, CA USA; 4 Department of Mathematics, University of California San Diego, San Diego, CA USA; 5 Computational and Applied Statistics Laboratory (CASL), SDSC – University of California, San Diego, CA USA; 6 Division of Gastroenterology, Hepatology, and Medicine, University of California San Diego, San Diego, California, USA

**Keywords:** Nonalcoholic fatty liver disease, Fibrosis, Elasticity imaging techniques, ROC curve, Magnetic resonance imaging

## Abstract

**Abstract:**

**Objectives:**

To assess reproducibility and fibrosis classification accuracy of magnetic resonance elastography (MRE)–determined liver stiffness measured manually at two different centers, and by automated analysis software in adults with nonalcoholic fatty liver disease (NAFLD), using histopathology as a reference standard.

**Methods:**

This retrospective, cross-sectional study included 91 adults with NAFLD who underwent liver MRE and biopsy. MRE-determined liver stiffness was measured independently for this analysis by an image analyst at each of two centers using standardized manual analysis methodology, and separately by an automated analysis. Reproducibility was assessed pairwise by intraclass correlation coefficient (ICC) and Bland-Altman analysis. Diagnostic accuracy was assessed by receiver operating characteristic (ROC) analyses.

**Results:**

ICC of liver stiffness measurements was 0.95 (95% CI: 0.93, 0.97) between center 1 and center 2 analysts, 0.96 (95% CI: 0.94, 0.97) between the center 1 analyst and automated analysis, and 0.94 (95% CI: 0.91, 0.96) between the center 2 analyst and automated analysis. Mean bias and 95% limits of agreement were 0.06 ± 0.38 kPa between center 1 and center 2 analysts, 0.05 ± 0.32 kPa between the center 1 analyst and automated analysis, and 0.11 ± 0.41 kPa between the center 2 analyst and automated analysis. The area under the ROC curves for the center 1 analyst, center 2 analyst, and automated analysis were 0.834, 0.833, and 0.847 for distinguishing fibrosis stage 0 vs. ≥ 1, and 0.939, 0.947, and 0.940 for distinguishing fibrosis stage ≤ 2 vs. ≥ 3.

**Conclusion:**

MRE-determined liver stiffness can be measured with high reproducibility and fibrosis classification accuracy at different centers and by an automated analysis.

**Key Points:**

• *Reproducibility of MRE liver stiffness measurements in adults with nonalcoholic fatty liver disease is high between two experienced centers and between manual and automated analysis methods.*

• *Analysts at two centers had similar high diagnostic accuracy for distinguishing dichotomized fibrosis stages.*

• *Automated analysis provides similar diagnostic accuracy as manual analysis for advanced fibrosis.*

**Supplementary Information:**

The online version contains supplementary material available at 10.1007/s00330-021-08381-z.

## Introduction

Magnetic resonance elastography (MRE) of the liver is increasingly used to noninvasively assess hepatic fibrosis in chronic liver disease [1, 2]. Prior studies have demonstrated the accuracy of MRE-determined liver stiffness for staging fibrosis [3–5], as well as its repeatability [6, 7].

To further advance the qualification of MRE-determined liver stiffness as a biomarker for liver fibrosis, it is important to establish reproducibility, which refers to the ability to obtain similar results despite changes in conditions and methodology in image acquisition and analysis [8]. Assessing reproducibility is essential for determining whether liver stiffness changes assessed at different centers or by different analysis methods can be attributed confidently to true change, rather than to measurement or analysis variability. It is also essential for determining whether and how measurements reported by different centers or described in publications using different analysis methods can be compared or pooled. Prior studies have examined inter-analyst reproducibility [7, 9] of MRE-determined liver stiffness measurements at the same center, but there is limited data on the reproducibility of measurements between centers. Such data is needed because agreement within any one analysis center may not generalize to agreement across other centers due to variability in training, experience, or other factors.

Published studies from different imaging and analysis centers consistently have found that MRE-derived liver stiffness is accurate compared to histopathology, but diagnostic cutoffs between studies have been inconsistent [10–14]. It is not clear whether these inconsistencies are due to differences in study population (e.g., fibrosis stage distribution, liver disease etiology, geographic or ethnic differences, body habitus), or to differences in measurement methodology. Manual measurement methods used in prior studies required drawing regions of interest (ROIs) that avoid artifact, major vessels, other organs, lesions, liver edges, and areas of poor wave propagation. Manually drawing such ROIs has unavoidable subjectivity, potentially introducing measurement variability. Patients with nonalcoholic fatty liver disease (NAFLD) are often obese and may be more difficult to image if they barely fit into the magnet bore [15]. To reduce potential sources of variability, an automated analysis method has been developed [16] and investigated in primary sclerosing cholangitis [17]. While this automated method should reduce or eliminate subjectivity and improve measurement reproducibility, it may not be as accurate as manual methods, and its agreement with manual measurement in patients with NAFLD is not fully understood.

Therefore, our primary purpose was to assess reproducibility, and fibrosis classification accuracy using histopathology as the reference, of 2D MRE-determined liver stiffness measured by automated and manual analysis at two centers in adults with NAFLD. Secondary purposes were to compare fibrosis classification accuracy and associated liver stiffness diagnostic cutoffs by automated and manual measurement at the two centers and to explore the effect of potential confounding factors upon reproducibility and accuracy.

## Material and methods

### Analysis design and subjects

This was a retrospective analysis of 2D MRE and liver biopsy data acquired prospectively in adults with known or suspected NAFLD at one clinical/imaging site and analyzed as part of this retrospective analysis at two analysis centers. Data for this analysis were compiled from three completed prospective parent studies, each HIPAA-compliant and approved by the institutional review board at the University of California, San Diego [18–20]. The subjects in the parent studies provided written informed consent. Eligibility criteria for the parent studies varied but required that the subject be ≥18 years of age and have known or suspected NAFLD [18, 19] or type 2 diabetes mellitus [20]. All parent studies excluded subjects with any of the following: liver disease other than NAFLD, substantial alcohol consumption (> 20 g/day for women or > 30 g/day for men), decompensated cirrhosis, or evidence of HCC. The final cohort consisted of 91 subjects from the three parent studies [18–20].

Consecutive subjects from the parent studies were retrospectively included in this analysis if they underwent liver biopsy to assess known or suspected NAFLD (for research or clinical care) and a standardized 2D MRE exam of the liver (for research) at center 1 between June 2011 and March 2015, within 180 days before or after liver biopsy. For subjects who had two or more MRE exams as part of a clinical trial, only the baseline MRE exam (before any intervention) was included. Subjects were excluded from this analysis if any of the following criteria were met: NAFLD treatment changed in the interim between MRE and liver biopsy, the MRE-biopsy time interval exceeded 180 days, MRE was technically inadequate (defined here as < 700 valid pixels cumulatively within all liver ROIs by at least two of the three MRE analysis methods), or missing MRE or fibrosis data. Ninety-one subjects meeting the above eligibility criteria were included in this analysis (Table 1).

**Table 1:** Subject demographics

Characteristic	Data (*n* = 91)
Sex	
Male (%) Female (%)	36 (39.6 %) 55 (60.4 %)
Adults	91 (100 %)
Age (y)	
Mean ± SD (range)	50.4 ± 14.3 (18.9–75.2)
BMI in adults (kg/m^2^)	
Mean ± SD	30.9 ± 5.1
Race	
White Hispanic Black Asian Hawaiian or pacific islander Native American Other / unknown	46 (50.5 %) 25 (27.5 %) 1 (1.1 %) 9 (9.9 %) 1 (1.1 %) 1 (1.1 %) 8 (8.8%)
Fibrosis	
0 (none) 1 (perisinusoidal or periportal) 2 (perisinusoidal and periportal) 3 (bridging fibrosis) 4 (cirrhosis)	47 (51.6 %) 22 (24.2 %) 7 (7.7 %) 10 (11.0 %) 5 (5.5 %)
Steatosis	
0 (< 5% hepatocytes) 1 (5–33% hepatocytes) 2 (33–66% hepatocytes) 3 (> 66% hepatocytes)	3 (3.3 %) 33 (36.3 %) 32 (35.2 %) 23 (25.3 %)
Lobular inflammation	
0 (no foci) 1 (< 2 foci per 200 × field) 2 (2–4 foci per 200 × field) 3 (> 4 foci per 200 × field)	4 (4.4 %) 46 (50.5 %) 38 (41.8 %) 3 (3.3 %)
Ballooning	
0 (no ballooned cells) 1 (few ballooned cells) 2 (many ballooned cells or prominent ballooning Missing	32 (35.2 %) 39 (42.9 %) 19 (20.9 %) 1 (1.1 %)
NASH diagnosis	
0 (not NAFLD) 1 (NAFLD) 2 (Borderline NASH) 3 (NASH) Missing	3 (3.3 %) 24 (26.4 %) 12 (13.2 %) 50 (54.9 %) 2 (2.2 %)
NAS	
0 1 2 3 4 5 6 7 8 N/A	1 (1.1 %) 1 (1.1 %) 10 (11.0 %) 16 (17.6 %) 23 (25.3 %) 16 (17.6 %) 16 (17.6 %) 4 (4.4 %) 1 (1.1 %) 3 (3.3 %)
MRI-PDFF by center 1 (%)	
Mean ± SD Range	16.3 ± 9.9 1.3–55.3
Manual 2D MRE stiffness by center 1 (kPa)	
Mean ± SD Range	3.06 ± 1.18 1.59–6.91
Manual 2D MRE stiffness by center 2 (kPa)	
Mean ± SD Range	3.12 ± 1.33 1.72–7.60
Automated 2D MRE stiffness (kPa)	
Mean ± SD Range	3.01 ± 1.16 1.69–7.02

Numbers in parentheses are percentages

*BMI* body mass index, *MRE* MR elastography, *NAFLD* nonalcoholic fatty liver disease,

NAS NAFLD Activity Score, *NASH* nonalcoholic steatohepatitis, *SD* standard deviation

All MRE examinations were analyzed independently for this analysis by one analyst at center 1, one analyst at center 2, and by an automated liver stiffness analysis method developed by center 2.

Demographics, height, weight at the time of MRE, time interval between MRE and biopsy, and biopsy results were recorded.

### MR examination

Subjects were examined supine at a single center 1 imaging site on a 3-T whole-body system (Signa Excite HDxt) with an eight-channel phased-array torso coil. A dielectric pad was placed between the coil and the abdominal wall. MR imaging and 2D MRE were performed in the same exam by MR technologists with a minimum of 1 year of experience performing MRE. The exam protocol included a chemical-shift-encoded MRI (CSE-MRI) technique to measure proton density fat fraction (PDFF) and *R*^2^* values. Whole-liver PDFF and *R*^2^* values were recorded. Image acquisition and calculation have been described in detail elsewhere [21, 22] and CSE-MRI image analysis is briefly described in Supplemental Materials. 2D MRE acquisition and reconstruction techniques are described in Supplemental Materials.

## Image analysis

### MR elastography standardized manual analysis

Standardized 2D MRE image analyses were performed by one experienced image analyst at each center (J.H. and M.Y.; 5 and 10 years of experience, respectively) using the MRE_Quant image analysis software (version 1.4, Mayo Clinic) for this analysis. ROI selection was performed on all slices on shear-wave images with simultaneous display of the magnitude image at the same level used for anatomical reference using the following standardized steps: (a) identification of liver parenchyma contour, excluding major vessels, and non-hepatic tissue; (b) exclusion of subcapsular liver tissue to avoid edge effects; (c) exclusion of multi-path wave interference and poor shear-wave amplitude; and (d) exclusion of areas identified by the inversion algorithm as invalid. Mean liver stiffness from all pixels in each ROI and the number of pixels in each ROI were recorded. A weighted mean liver stiffness was computed over all pixels for each exam.

### MR elastography automated analysis

Automated 2D MRE image analysis was supervised by an engineer (B.D., 9 years of experience) at center 2, blinded to and independent of the manual analyses performed at each center, using the automated liver elasticity calculation (ALEC) algorithm (Mayo Clinic [16]). The mean stiffness and number of pixels in the cumulative ROIs drawn by the automated algorithm were recorded.

**Fig. 1:**
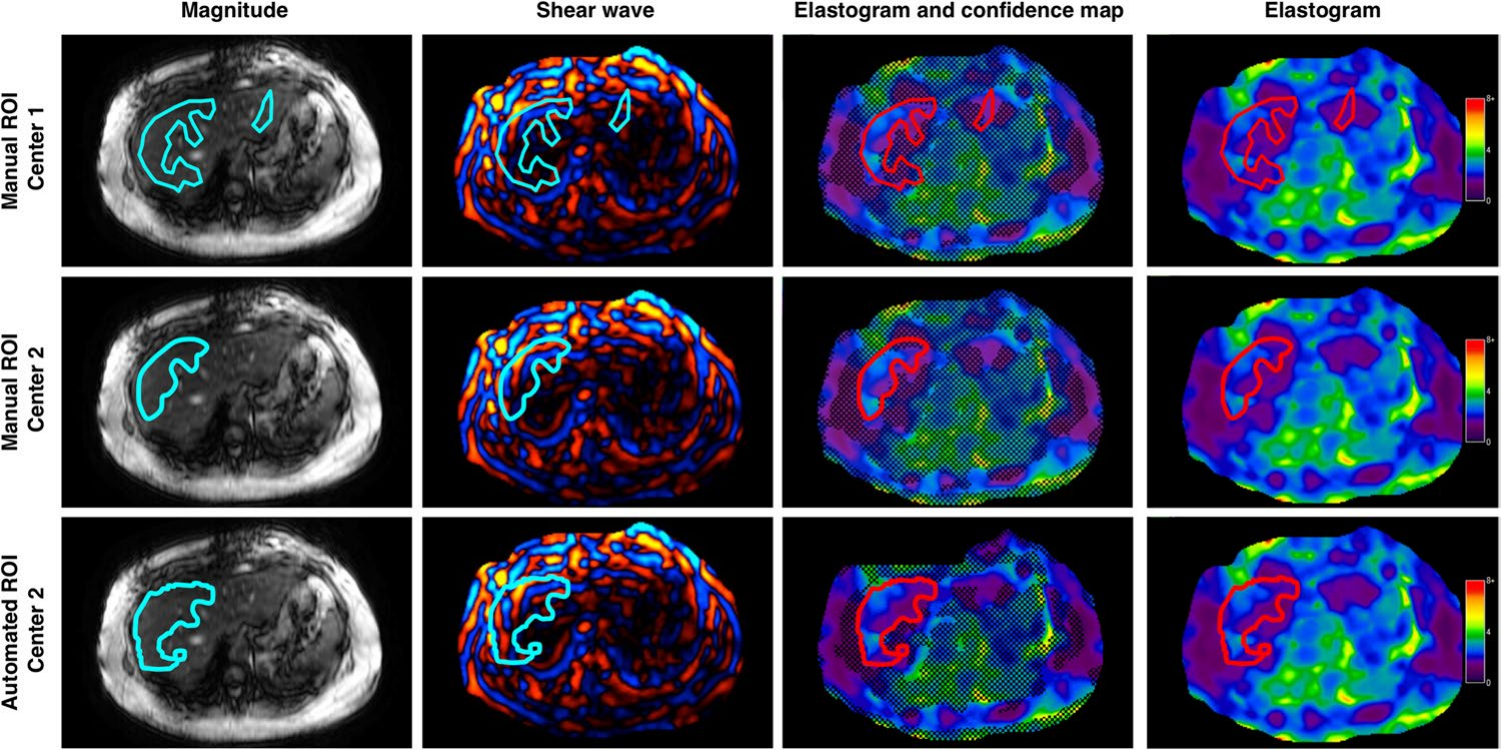
A 49-year-old woman with biopsy-determined diagnosis of NASH. The four columns correspond to axial magnitude images used for anatomical reference, shear-wave images, elastogram images with a parametric map of goodness-of-fit represented as hatched areas with ROIs, and elastograms without confidence maps. The three rows show regions of interests drawn manually at center 1, manually at center 2, and automated analysis at center 2 indicating a stiffness of 2.01 ± 0.44 kPa, 2.07 ± 0.43 kPa, and 1.93 ± 0.44 kPa and ROI sizes of 857 pixels, 920 pixels, and 1902 pixels, respectively. *ROI* region of interest

An example of elastograms with ROIs drawn manually at each center, and the automated analysis is shown in Fig. 1.

### Liver biopsy and histopathology scoring

Liver biopsy and histopathology scoring methods according to the NASH Clinical Research Network histologic system [23] are described in Supplemental Materials.

### Blinding

The image analysts drawing ROIs or the engineer supervising automated MRE image analysis were blinded to histopathological results. The hepatopathologists were blinded to the MRE results.

### Statistical analysis

Statistical analyses were performed with software package (R version 3.5.1 (2018); R: A language and environment for statistical computing. R Foundation for Statistical Computing). Demographics, histopathologic scoring, and imaging features were summarized. Categorical variables were expressed as numbers and percentages. Continuous variables were expressed as mean (± SD).

#### Reproducibility

Measurement reproducibility of 2D MRE-determined liver stiffness by the three MRE analysis methods (center 1 analyst, center 2 analyst, automated) was assessed pairwise using the Bland-Altman analysis. Metrics recommended by the Quantitative Imaging Biomarkers Alliance ® (QIBA) [24] were obtained and presented: intraclass correlation coefficient (ICC), bias, standard deviation (SD), limits of agreement (LOA), within-subject coefficient of variation (wCV), and coefficient of reproducibility (RDC). Bland-Altman plots and scatterplots were generated. ICC-based agreement was interpreted according to the Landis and Koch scale [25]. In addition to pairwise analyses, ICC was computed overall for the three sets of MRE-determined liver stiffness measurements.

#### Accuracy

Spearman's correlation coefficient between MRE-determined liver stiffness measured by each of the three MRE analysis methods, and fibrosis stage was computed. Correlations were compared using Williams’ test for two dependent correlations sharing one variable [26].

Classification accuracy of MRE-determined liver stiffness measured by each of the three MRE analysis methods was assessed for distinguishing fibrosis stage 0 vs. 1–4 (i.e., diagnosing the presence of any fibrosis) and for distinguishing fibrosis stages ≤ 2 vs. ≥ 3 (i.e., diagnosing the presence of advanced fibrosis). These thresholds were selected in part by their importance (presence of any fibrosis and presence of advanced fibrosis are usually of specific interest), and in part by our data distribution. In particular, there were not enough subjects with stage 2 or stage 4 fibrosis to meaningfully examine ≤ 1 vs. ≥ 2 or ≤ 3 vs. 4 thresholds, respectively. For each set of dichotomized fibrosis stages, the area under the receiver operating characteristic curve (AUC) was calculated. The thresholds that provided the best sensitivity at ≥ 90% specificity to differentiate dichotomized fibrosis stages were selected. Emphasis was placed on high specificity to assess the performance of MRE as a potential rule-in test, a context of use in which high specificity is needed. For each threshold, raw and cross-validated sensitivity, specificity, accuracy, positive predictive value (PPV), and negative predictive value (NPV) were calculated. Exact binomial and bootstrap 95% confidence intervals (CIs) were computed around each performance parameter or AUC estimate, respectively.

#### Secondary analyses

AUC, sensitivity, specificity, and total accuracy of MRE-determined liver stiffness measured by the three MRE analysis methods were compared pairwise using bootstrap-based tests. Piece-wise Bonferroni correction for multiple comparisons was applied to each set of comparisons (classification of the presence of fibrosis was treated as one set, and classification of advanced fibrosis was treated as another set). Within each set, there were twelve comparisons (ROC AUC and sensitivity, specificity, total accuracy compared pairwise between the three MRE methods); thus, *p* values of 0.0042 (i.e., 0.05/12) or less were considered statistically significant to ensure a family-wise 0.05 significance level.

The analysis of potential confounding effects on reproducibility and accuracy is described in Supplemental Materials.

## Results

### Cohort characteristics

Cohort characteristics are summarized in Table 1 (55 female, 36 male; age range 18.9 to 75.2 years). The mean and standard deviation of BMI were 31.3 ± 5.1 kg/m^2^. Forty-seven, 22, 7, 10, and 5 subjects had fibrosis stage 0, 1, 2, 3, and 4, respectively. Eighty-eight of 91 subjects (97%) had at least grade 1 steatosis. The mean and standard deviation of PDFF were 16.3% ± 9.9%, with a range from 1.3 to 55.3%. Mean and standard deviation of 2D MRE-determined liver stiffness by center 1 analyst, center 2 analyst, and automated analysis were 3.06 ± 1.18 kPa, 3.12 ± 1.33 kPa, and 3.01 ± 1.16 kPa, respectively. Across all three analysis methods, MRE-determined liver stiffness ranged from 1.59 to 7.60 kPa.

The mean time interval between MRE and biopsy was 47 days (interquartile range: 15 to 63 days). Three MRE exams were considered inadequate due to ROI size < 700 Px by at least two of the three MRE analysis methods: two had inadequate ROI size by both analysts, while the third had inadequate ROI size by the center 2 analyst and the automated analysis. All other exams with one exception had ROI size > 700 Px by all three MRE analysis methods. The exception was an exam with ROI size of 688 Px by the center 1 analyst, but > 700 Px by the other two analysis methods.

### Reproducibility

**Fig. 2:**
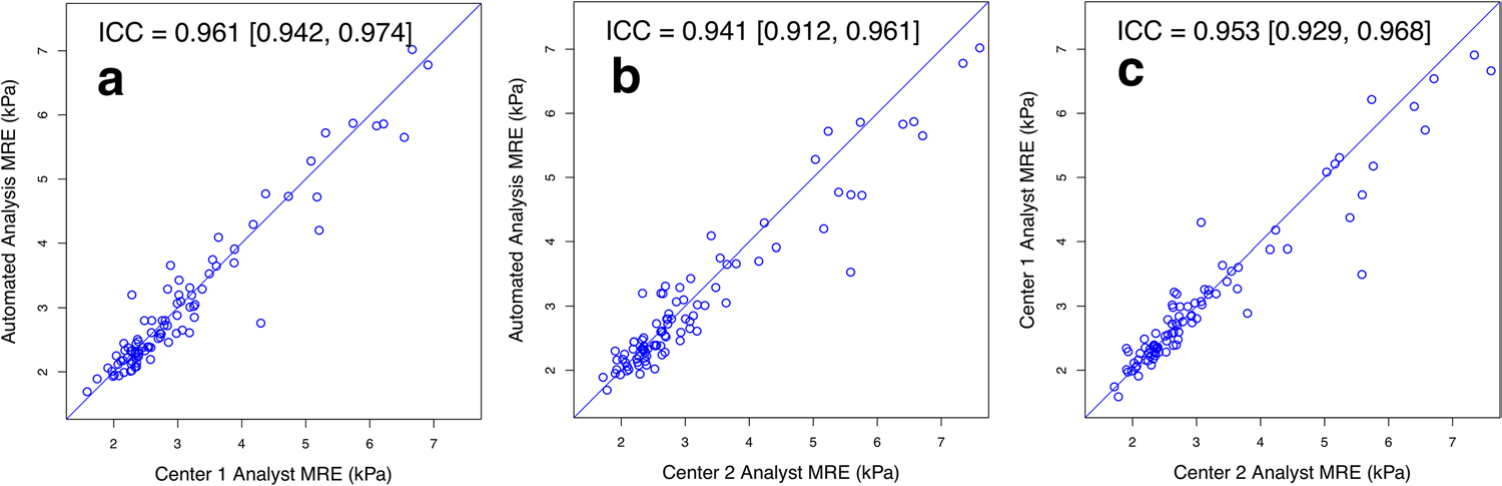
Scatterplots of liver stiffness estimates: **a** Center 1 analyst vs. automated analysis. **b** Center 2 analyst vs. automated analysis. **c** Center 1 analyst vs. center 2 analyst

All ICCs were in the excellent range. As summarized in Fig. 2 and Table 2, pairwise ICCs ranged from 0.941 to 0.961, depending on the comparison. Overall ICC (95% confidence interval [CI]) was 0.951 (0.932, 0.966).

**Table 2:** Inter-analysis method reproducibility of 2D MRE stiffness estimates

Analysis method comparison	ICC (95% CI)	Bland-Altman analysis		wCV	RDC
		Bias ± SD [kPa]	95% LOA [kPa]		
Center 1 analyst vs. automated analysis	0.961 (0.942–0.974)	0.05 ± 0.32 (*p* = 0.133)	−0.58, 0.69	7.0%	19.5%
Center 2 analyst vs. automated analysis	0.941 (0.912–0.961)	0.11 ± 0.41 (*p* < 0.001)	−0.70, 0.93	8.2%	22.8%
Center 1 analyst vs. center 2 analyst	0.953 (0.929–0.968)	0.06 ± 0.38 (*p* = 0.119)	−0.69, 0.82	7.1%	19.6%
Overall	0.951 (0.932–0.966)	–	–	–	–

**Fig. 3:**
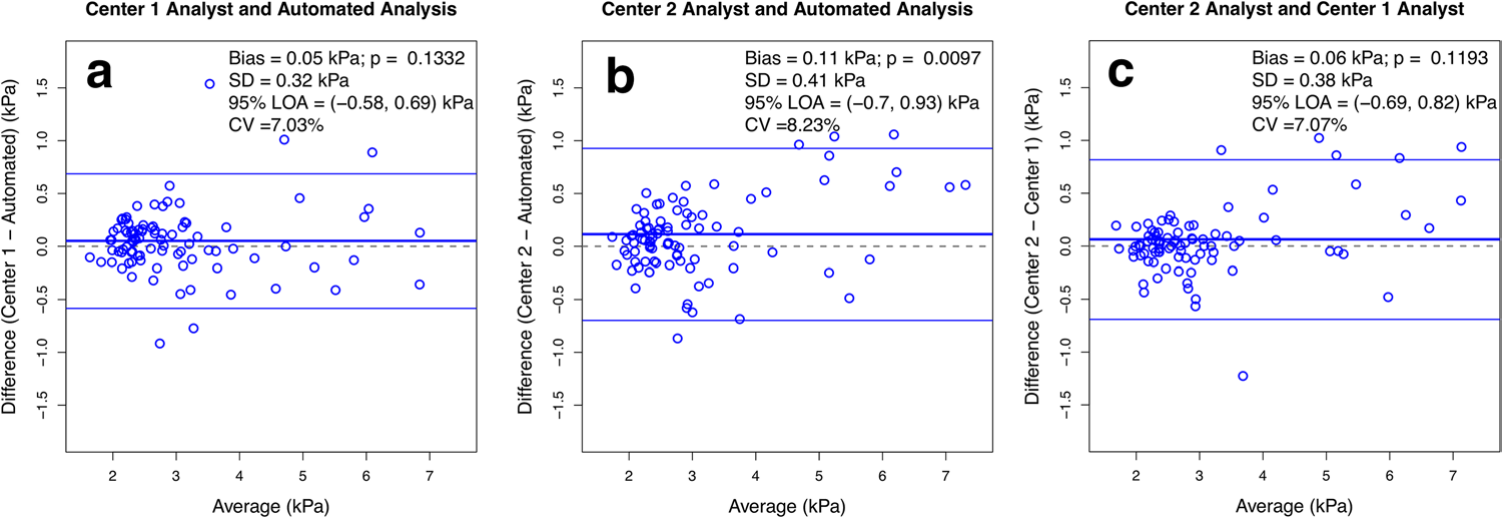
Bland-Altman plots. **a** Center 1 analyst vs. automated analysis. **b** Center 2 analyst vs. automated analysis. **c** Center 2 analyst vs. center 1 analyst

The Bland-Altman analysis showed good agreement between analysts at each center and between methods (Fig. 3, Table 2). The bias (0.11 kPa) between mean MRE-determined liver stiffness measured by the center 2 analyst vs. automated analysis was statistically significant (*p* = 0.0097); however, the biases between the center 1 analyst vs. automated analysis (0.05 kPa) and between the analysts at each center (0.06 kPa) were not significant (*p* = 0.1332 and *p* = 0.1193, respectively). RDC was 19.5% between the center 1 analyst and automated analysis, 22.8% between center 2 analyst and automated analysis, and 19.6% between the analysts at each center.

### Accuracy

**Fig. 4:**
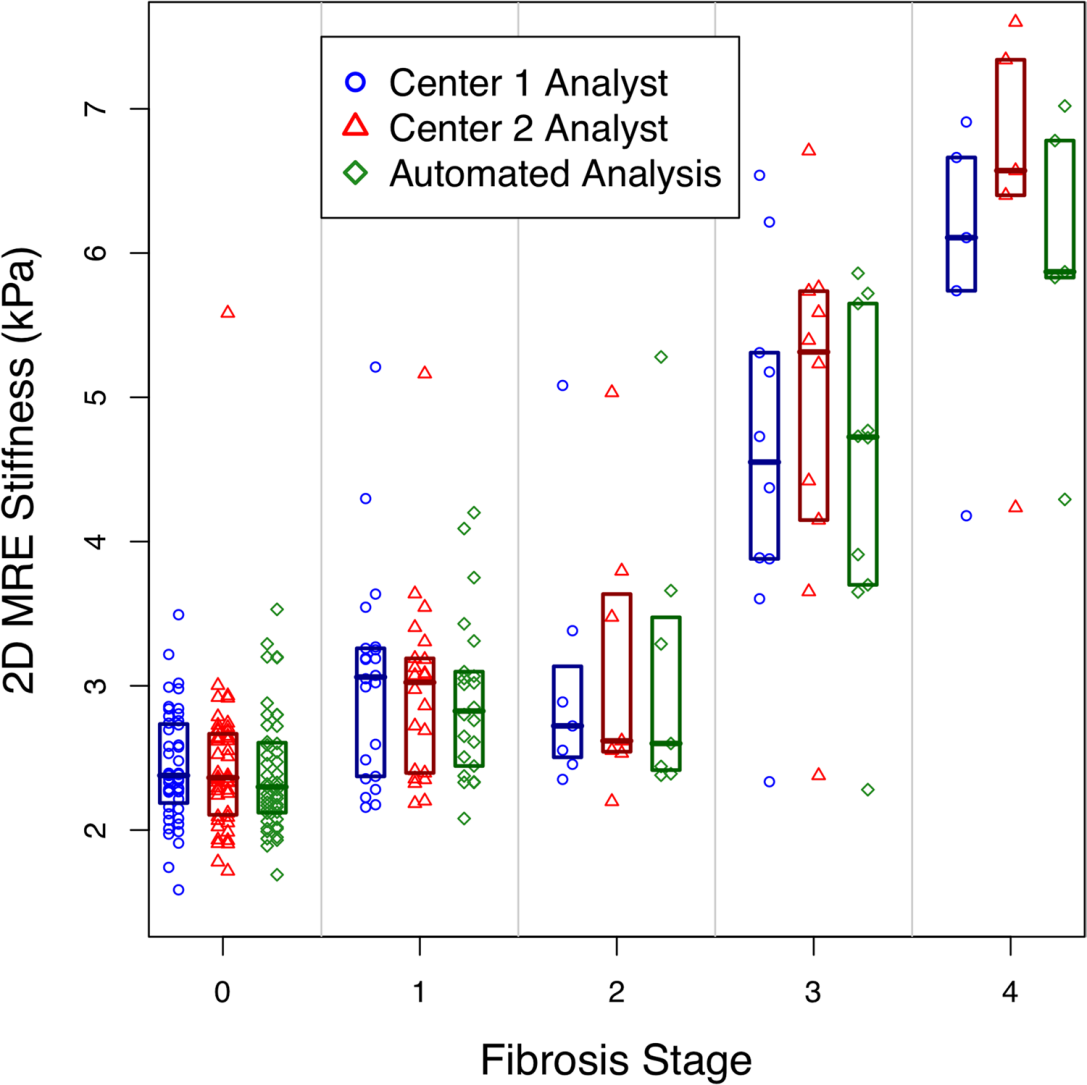
Scatterplot with superimposed boxplots shows 2D MRE stiffness estimated in kPa vs. liver fibrosis stages for center 1 analyst, center 2 analyst, and automated analysis

Spearman correlation coefficients between MRE-determined liver stiffness measurements and histopathology were positive (Fig. 4), and statistically significant for all three analysis methods (center 1 analyst: Spearman’s *ρ* = 0.648, *p* < 0.0001; center 2 analyst: Spearman’s *ρ* = 0.650, *p* < 0.0001; automated analysis: Spearman’s *ρ* = 0.670, *p* < 0.0001). Pairwise differences in Spearman correlations were not significant (*p* > 0.4 for all comparisons).

**Fig. 5:**
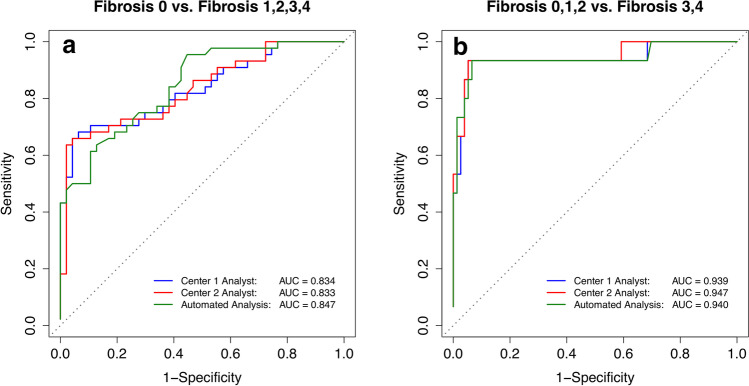
Receiver operating characteristic curve analysis of MRE-stiffness measured by center 1 analyst, center 2 analyst and automated analysis for classification of dichotomized fibrosis stages using histology as a reference standard: **a** 0 versus ≥ 1 and **b** ≤ 2 versus ≥ 3. The corresponding area under the curves is indicated in the graphs

As summarized in Table 3 and Fig. 5, AUCs of the center 1 analyst, center 2 analyst, and automated analysis were 0.834, 0.833, and 0.847 for distinguishing fibrosis stage 0 vs. ≥ 1 and 0.939, 0.947, and 0.940 for distinguishing fibrosis stage ≤ 2 vs. ≥ 3. The corresponding MRE-stiffness (Fig. 5) cutoffs that provided the best sensitivity at ≥ 90% specificity were 2.99, 2.98, and 3.29 kPa for distinguishing fibrosis stage 0 vs. ≥ 1 and 3.60, 3.65, and 3.65 kPa for distinguishing fibrosis stage ≤ 2 vs. ≥ 3. At those cutoffs, raw sensitivities of the center 1 analyst, center 2 analyst, and automated analysis were 68%, 66%, and 50% and 93%, 93%, and 93% for the two histologic classifications. Raw specificities of the center 1 analyst, center 2 analyst, and automated analysis were 94%, 96%, and 96% and 95%, 95%, and 93% for the two histologic classifications. Cross-validated estimates are reported in Supplemental Table 1.

**Table 3:** Raw classification accuracy parameters of 2D MRE for staging liver fibrosis

Fibrosis stage classification	ROC area	MRE stiffness cutoff	Sensitivity (%)	Specificity (%)	Accuracy (%)	PPV (%)	NPV (%)
Center 1 analyst							
0 (*n* = 58) vs. ≥ 1 (*n* = 33)	0.834 [0.734, 0.912]	2.99 kPa	68 (30/44) [52, 81]	94 (44/47) [82, 99]	81 (74/91) [72, 89]	91 (30/33) [76, 98]	76 (44, 58) [63, 86]
≤ 2 (*n* = 73) vs. ≥ 3 (*n* = 18)	0.939 [0.843, 0.997]	3.60 kPa	93 (14/15) [68, 100]	95 (72, 76) [87, 99]	95 (86, 91) [88, 98]	78 (14, 18) [52, 94]	99 (72, 73) [93, 100]
Center 2 analyst							
0 (*n* = 60) vs. ≥ 1 (*n* = 31)	0.833 [0.745, 0.912]	2.98 kPa	66 (29/44) [50, 80]	96 (45/47) [85, 99]	81 (74/91) [72, 89]	94 (29, 31) [79, 99]	75 (45/60) [62, 85]
≤ 2 (*n* = 73) vs. ≥ 3 (*n* = 18)	0.947 [0.856, 0.997]	3.65 kPa	93 (14/15) [68, 100]	95 (72, 76) [87, 99]	95 (86, 91) [88, 98]	78 (14, 18) [52, 94]	99 (72, 73) [93, 100]
Automated analysis							
0 (*n* = 67) vs. ≥ 1 (*n* = 24)	0.847 [0.765, 0.915]	3.29 kPa	50 (22/44) [35, 65]	96 (45/47) [85, 99]	74 (67/91) [67, 78]	92 (22/44) [73, 99]	67 (45/67) [55, 78]
≤ 2 (*n* = 72) vs. ≥ 3 (*n* = 19)	0.940 [0.834, 0.997]	3.65 kPa	93 (14/15) [68, 100]	93 (71/76) [85, 98]	93 (85/91) [86, 98]	74 (14/19) [49, 91]	99 (71/72) [93, 100]

*n* = number of subjects in each dichotomized fibrosis stage. Data in parentheses are raw data, and data in brackets are 95% confidence intervals.

*ROC* receiver operating characteristic, *PPV* positive predictive value, *NPV* negative predictive value, *MRE* magnetic resonance elastography

### Secondary analyses

#### Accuracy comparisons

Pairwise comparisons of performance parameters for classifying the presence of any fibrosis and of advanced fibrosis are reported in Supplemental Table 2. Both the center 1 analyst and the center 2 analyst had higher sensitivity (*p* < 0.001 and *p* < 0.001, respectively, significant at 0.05 level after Bonferroni’s adjustment) and accuracy (*p* = 0.002 and *p* = 0.004, respectively, significant at 0.05 level after Bonferroni’s adjustment) than the automated analysis for distinguishing fibrosis stage 0 vs. ≥ 1, but not for distinguishing fibrosis stage ≤ 2 from ≥ 3 (*p*: 0.300–0.950). Center 1 analyst and center 2 analyst were not different from one another on any of the performance parameters (*p*: 0.190–0.950).

#### Confounders of reproducibility and accuracy

These are presented in Table 4*.*

**Table 4:** Confounders of accuracy. Pairwise comparisons of performance parameters for classifying the presence of significant fibrosis and advanced fibrosis. *p* values of 0.0042 (0.05/12) or less can be considered significant at the family-wise 0.05 level after the Bonferroni adjustment (highlighted in bold in this table)

Performance metric	Comparison	Fibrosis stage	Estimate	*p* value
AUC	Center 1 analyst vs. automated analysis	0 vs ≥ 1	0.001 (−0.045, 0.052)	0.95
		≤ 2 vs ≥ 3	−0.008 (−0.049, 0.016)	0.30
	Center 1 analyst vs. center 2 analyst	0 vs ≥ 1	−0.013 (−0.091, 0.050)	0.51
		≤ 2 vs ≥ 3	−0.001 (−0.033, 0.018)	0.71
	Center 2 analyst vs. automated analysis	0 vs ≥ 1	−0.014 (−0.091, 0.053)	0.58
		≤ 2 vs ≥ 3	0.007 (−0.017, 0.050)	0.41
Sensitivity	Center 1 analyst vs. automated analysis	0 vs ≥ 1	0.182 (0.024, 0.395)	**0.001**
		≤ 2 vs ≥ 3	0.000 (−0.385, 0.000)	0.95
	Center 1 analyst vs. center 2 analyst	0 vs ≥ 1	0.023 (−0.152, 0.183)	0.24
		≤ 2 vs ≥ 3	0.000 (−0.385, 0.000)	0.95
	Center 2 analyst vs. automated analysis	0 vs ≥ 1	−0.433 (−0.739, −0.181)	**0.001**
		≤ 2 vs ≥ 3	0.000 (−0.467, 0.000)	0.95
Specificity	Center 1 analyst vs. automated analysis	0 vs ≥ 1	−0.021 (−0.100, 0.053)	0.200
		≤ 2 vs ≥ 3	0.013 (−0.069, 0.065)	0.44
	Center 1 analyst vs. center 2 analyst	0 vs ≥ 1	−0.021 (−0.100, 0.059)	0.190
		≤ 2 vs ≥ 3	0.000 (−0.096, 0.043)	0.36
	Center 2 analyst vs. automated analysis	0 vs ≥ 1	0.010 (−0.058, 0.099)	0.36
		≤ 2 vs ≥ 3	−0.013 (−0.068, 0.063)	0.40
Accuracy	Center 1 analyst vs. automated analysis	0 vs ≥ 1	0.077 (0.000, 0.198)	**0.002**
		≤ 2 vs ≥ 3	0.011 (−0.066, 0.044)	0.62
	Center 1 analyst vs. center 2 analyst	0 vs ≥ 1	0.000 (−0.121, 0.044)	0.450
		≤ 2 vs ≥ 3	0.000 (−0.077, 0.037)	0.40
	Center 2 analyst vs. automated analysis	0 vs ≥ 1	−0.077 (−0.187, 0.000)	**0.004**
		≤ 2 vs ≥ 3	−0.011 (−0.062, 0.044)	0.47

## Discussion

This retrospective analysis of prospectively acquired data assessed inter-center and inter-method analysis reproducibility of 2D MRE-determined liver stiffness measurement in adults with suspected or known NAFLD and explored possible covariate effects. Using a standardized manual image analysis method, we found that MRE-determined liver stiffness measured independently by analysts at two centers had high reproducibility with each other and with an automated analysis method, with pairwise biases of ≤ 0.11 kPa, ICCs of ≥ 0.941, and RDCs of ≤ 22.8%.

In a study performed in a Japanese population, Motosugi et al [6] found a nearly perfect intraclass correlation coefficient and bias of 0.03 kPa between two readers. The reproducibility observed in our analysis is slightly lower, which is to be expected since our analysis incorporated an additional source of variability (center effect), did not include normal volunteers, and focused on the NAFLD population, which can be more challenging to cover the entire organ and minimize ROI select bias. In a longitudinal study, Hines et al [7] examined the reproducibility of MRE stiffness estimates on the same day and 2–4 weeks later in 20 healthy volunteers and in 10 patients with mixed liver conditions. The authors attributed variability to diurnal physiological changes (8.5%), replicate exams on the same day (4.2%), inter-reader variability (1.9%), and intra-reader variability (1.4%). Our results revealed higher inter-reader wCVs (7.0 to 8.2%) than the magnitude of inter-reader variability observed by Hines et al [7], albeit with a substantially larger sample size and exclusive enrollment of subjects with known or suspected NAFLD.

Liver stiffness estimates from analysts at both centers had similar classification accuracy for distinguishing dichotomized fibrosis stage, with high ROCs (≥ 0.833 for fibrosis stage vs. ≥ 1; ≥ 0.939 for fibrosis stage ≤ 2 vs. ≥ 3) similar to previous reports in adults with NAFLD [1, 14, 27–30], and virtually identical diagnostic thresholds. Our analysts’ thresholds (2.99 kPa and 2.98 kPa for fibrosis stage 0 vs. ≥ 1, and 3.60 kPa and 3.65 kPa for fibrosis stage ≤ 2 vs. ≥ 3) were similar to those reported by Loomba et al (31) in a previous study using the same MRE acquisition and inversion technique at the same field strength in patients with NAFLD and NASH: 3.02 kPa for distinguishing fibrosis stage 0 vs. ≥ 1 and 3.77 kPa for distinguishing stage ≤ 2 vs. ≥ 3 [28]. The automated analysis method provided similar performance for fibrosis stage ≥ 3 with comparable thresholds and diagnostic parameters. However, the automated method had a slightly higher diagnostic cutoff for fibrosis stage 0 vs. ≥ 1 with correspondingly lower sensitivity.

We also examined potential confounders that could affect inter-center and inter-analysis reproducibility and accuracy in our cohort. We found no confounders for reproducibility. However, we found that higher BMI was associated with classification errors for fibrosis stage ≤ 2 vs. ≥ 3 by all three analysis methods, and higher PDFF was associated with classification errors for fibrosis stage 0 vs. ≥ 1 by two out of three analysis methods. No other covariate among those examined affected the classification accuracy. These results suggest that larger patient body habitus and severity of liver steatosis affect the classification accuracy of MRE for fibrosis staging.

Inter-center RDC (19.6%) in our analysis was similar to that reported by the QIBA MRE Committee in a meta-analysis of test-retest repeatability studies acquired from various imaging centers [31]. The QIBA claim is that “measured change in hepatic stiffness of 19% or larger indicates that a true change in stiffness has occurred with 95% confidence” [32]. However, our analysis had a fundamentally different design than the QIBA meta-analysis, and the interpretation of our results is complementary and non-redundant. Unlike the meta-analysis, our analysis utilized MRE data acquired cross-sectionally in a standardized fashion from a single imaging center in clinical patients with NAFLD and incorporated the effect of analysis center on reproducibility. Our inter-center RDC suggests that if MRE-determined liver stiffness in adults with NAFLD is assessed by experienced analysts at different centers, 95% of the liver stiffness estimates will agree within 19.6%. Furthermore, the liver stiffness estimates made by analysts at the two centers were not systematically different (bias = 0.06 kPa, *p* = 0.1193), about an order of magnitude smaller than the classification cutoffs (~3 to 3.6 kPa)—and unlikely to be meaningful in clinical care or most clinical trials. The coefficient of variation between analysis centers was also low (7.1%) and introduced less variability than diurnal physiological changes reported previously (8.5%) [7]. Additionally, diagnostic cutoffs for our two centers (2.99 kPa and 2.98 kPa for fibrosis stage ≥ 1; 3.60 and 3.65 kPa for fibrosis stage ≥ 3) were virtually identical. Taken together, these results suggest that a standardized manual MRE analysis method provides excellent inter-center reproducibility and that measurements made at different analysis centers are comparable. Moreover, these results provide indirect but independent support that MRE-determined liver stiffness measurements by experienced analysts in different published studies can be pooled and that inconsistency in published diagnostic cutoffs probably reflects factors other than variability in how the MRE data was measured.

RDCs between manual and automated analysis were 19.5% and 22.8%, for our two analysts, suggesting that whether MRE-determined liver stiffness is estimated manually or by automated analysis, 95% of the estimates will agree within ≤ 22.8%. In combination with the other results of our analysis, this finding provides preliminary evidence that an automated analysis method performs about as well as experienced analysts in measuring MRE-determined liver stiffness, at least for purposes of diagnosing advanced fibrosis in adults with NAFLD. Further research is needed to more fully validate this promising automated analysis methodology.

Strengths of our analysis included the high quality of the MRE data, which was obtained by experienced MRI technologists using a standardized acquisition protocol and measured independently by experienced analysts at two analysis centers. Also, the selective inclusion of subjects with NAFLD without healthy volunteers makes our results relevant to MRE technical performance in the NAFLD population.

Our analysis had limitations. First, we only compared MRE-determined liver stiffness measurements by one experienced analyst at each of two academic analysis centers. Future research is required to examine inter-center reproducibility across a larger number and a broader spectrum of analysis centers, including community settings and non-expert analysts. Second, the distribution of our sample across fibrosis stages was not balanced and we did not have a sufficient number of patients with fibrosis stages 2 or 4 to allow investigation of all histologic classifications. Third, we were not able to assess the effect of field strength, type of MRE sequence, MRI manufacturer, or MRI system as potential sources of variability in MRE-determined liver stiffness estimates because all exams in this analysis were acquired on a single (3T GE) scanner) with a 2D GRE sequence and analyzed with a 2D inversion algorithm. 2D spin-echo echo-planar MRE sequences have been shown to be superior to 2D GRE MRE sequences at 3T [33], but those sequences were not available to us at the time of the parent studies for this analysis. Further studies systematically addressing these additional sources of variability are needed. Fourth, we used a 700-pixel cutoff for total ROI area across four slices acquired through the widest part of the liver as a conservative threshold indication of technical MRE analyzability reported elsewhere [34]. While this cutoff may appear small, the criteria for valid measurement remain an active area for investigation by QIBA. Further research is needed to identify whether total ROI area (instead of pixel numbers) is the best way to assess MRE analyzability and to determine the minimal cutoff that provides satisfactory accuracy and precision for various contexts of use, including diagnostic enrichment, disease stratification, treatment prediction, and treatment response assessment. Finally, our analysis was not designed to and did not evaluate the severity of any misclassifications that may have occurred. Future studies should include this type of analysis to help ensure that proposed analysis methodologies do not introduce clinically unacceptable misclassifications.

In conclusion, our retrospective analysis of previously acquired liver 2D MRE exams performed on the same 3-T scanner at a single center indicate high, but imperfect inter-center and inter-analysis method agreement of MRE-determined liver stiffness. MRE fibrosis classification thresholds and accuracy were similar for analysts at two analysis center, and an automated analysis method provided similar performance to manual analysis for advanced fibrosis but may have lower sensitivity for detecting any fibrosis.

## Supplementary Information

ESM 1 (DOCX 50 kb)

## Abbreviations

AUC: Area under the receiver operating characteristic curve
BIC: Bayesian information criterion
BMI: Body mass index
GRE: Gradient-recalled echo
ICC: Intraclass correlation coefficient
kPa: Kilopascal
LOA: Limits of agreement
MRE: Magnetic resonance elastography
MRI: Magnetic resonance imaging
NAFLD: Nonalcoholic fatty liver disease
NAS: NAFLD Activity Score
NASH: Nonalcoholic steatohepatitis
NPV: Negative predictive value
PDFF: Proton density fat fraction
PPV: Positive predictive value
Px: Pixel
QIBA: Quantitative Imaging Biomarker Alliance
RDC: Reproducibility coefficient
ROC: Receiver operating characteristic
ROI: Region of interest
SD: Standard deviation
wCV: Within-subject coefficient of variation

## Declarations

### Guarantor

The scientific guarantor of this publication is Dr. Claude B. Sirlin.

### Statistics and biometry

Tanya Wolfson, Lin Zheng, and Anthony Gamst are biostatisticians. Tanya Wolfson and Lin Zheng performed statistical analysis of the data under the supervision of Anthony Gamst. Tanya Wolfson controlled the data. Tanya Wolfson, Lin Zheng, and Anthony Gamst are not employed by or are consultants for any company in the medical industry.

### Study subjects or cohorts overlap

The MRE and liver biopsy data for this retrospective analysis is compiled from three separate prior prospective, HIPAA-compliant, IRB-approved studies in adults with known or suspected NAFLD performed at UCSD between June 2011 and March 2015. Those studies were individually reported in 3 publications (references below and PDFs attached to the current submission).

Park CC, Nguyen P, Hernandez C, Bettencourt R, Ramirez K, Fortney L, Hooker J, Sy E, Savides MT, Alquiraish MH, Valasek MA, Rizo E, Richards L, Brenner D, Sirlin CB, Loomba R. Magnetic resonance elastography vs transient elastography in detection of fibrosis and noninvasive measurement of steatosis in patients with biopsy-proven nonalcoholic fatty liver disease. *Gastroenterology*. 2017 Feb;152(3):598-607.e2. doi: 10.1053/j.gastro.2016.10.026. Epub 2016 Oct 27. PMID: 27911262; PMCID: PMC5285304.

Loomba R, Sirlin CB, Ang B, Bettencourt R, Jain R, Salotti J, Soaft L, Hooker J, Kono Y, Bhatt A, Hernandez L, Nguyen P, Noureddin M, Haufe W, Hooker C, Yin M, Ehman R, Lin GY, Valasek MA, Brenner DA, Richards L; San Diego Integrated NAFLD Research Consortium (SINC). Ezetimibe for the treatment of nonalcoholic steatohepatitis: assessment by novel magnetic resonance imaging and magnetic resonance elastography in a randomized trial (MOZART trial). *Hepatology*. 2015 Apr;61(4):1239-50. doi: 10.1002/hep.27647. Epub 2015 Feb 27. PMID: 25482832; PMCID: PMC4407930.

Doycheva I, Cui J, Nguyen P, Costa EA, Hooker J, Hofflich H, Bettencourt R, Brouha S, Sirlin CB, Loomba R. Non-invasive screening of diabetics in primary care for NAFLD and advanced fibrosis by MRI and MRE. *Aliment Pharmacol Ther.* 2016 Jan;43(1):83-95. doi: 10.1111/apt.13405. Epub 2015 Sep 15. PMID: 26369383; PMCID: PMC4673036.

In this analysis, we address knowledge gaps in adults with NAFLD not investigated or reported in the parent studies: (1) 2D MRE stiffness measurement reproducibility between different centers and between manual and automated analysis methods; (2) comparison of diagnostic accuracy for distinguishing dichotomized fibrosis stages between different centers; and (3) comparison of diagnostic accuracy for distinguishing dichotomized fibrosis stages between manual analysis and automated analysis.

### Methodology

• Retrospective

• Cross-sectional study

• Multicenter study
