# Supplementary material for: MR elastography in nonalcoholic fatty liver disease: inter-center and inter-analysis-method measurement reproducibility and accuracy at 3T
Source: Eur Radiol. 2021 Dec 20;32(5):2937–48. doi: 10.1007/s00330-021-08381-z (PMC9038857; doi:10.1007/s00330-021-08381-z)
Supplement: Supplementary file 1 — (DOCX 50 kb) [file 330_2021_8381_MOESM1_ESM.docx]

**SUPPLEMENTAL MATERIAL**

*CSE-MRI image analysis:* Using the software OsiriX (v5.8; Geneva, Switzerland), an image analyst (J.H.; 5 years of experience) in Center 1 at University of California, San Diego drew circular regions of interest (ROIs) of 20 mm in diameter in each of the nine Couinaud liver segments on parametric PDFF and R2* maps for each subject, for this analysis. Mean PDFF and R2* values from the nine ROIs were recorded.

*MR elastography:* A 19-cm diameter, 1.5 cm thick acoustic pressure-activated passive driver (Resoundant Technology, Rochester, MN) was placed under the dielectric pad and against the right anterior chest wall with its center level with the xiphoid process at the midclavicular line. An active driver device located outside the scanner room transmitted acoustic longitudinal pressure waves to the passive driver at a frequency of 60 Hz. These longitudinal waves are known to generate shear waves throughout the abdomen including the liver by the mode conversion phenomenon at tissue interfaces [[2](#_ENREF_2)].

To visualize the generated shear waves, 2D MRE raw images were obtained with a GRE phase-contrast sequence through the widest part of the liver. Four axial images were acquired with a 50 ms TR, 20 ms TE) 30° flip angle, 10 mm slice thickness, 256 x 64 matrix, ± 30 kHz bandwidth, 2x parallel imaging acceleration factor, one pair of motion-encoding gradients with zero and first gradient-moment nulling, and four 90° phase offsets between the mechanical vibration and the motion encoding gradients. The total acquisition time for this four-slice MRE acquisition was 56 s, divided into four 14-s breath-holds [[2-4](#_ENREF_2)]. Wave images were processed with a two-dimensional (2D) multi-model direct inversion algorithm to generate quantitative images displaying shear stiffness (MR elastograms) [[5](#_ENREF_5)]. The inversion algorithm used to calculate stiffness from the wave images uses the MRE displacement data in sliding windows to perform a direct inversion of the differential equation modeling the wave propagation. A second- or fourth-order polynomial is fit to the data depending on fit quality. The correlation coefficient for the fit is recorded for the center pixel of each window. This results in a confidence map with values between 0 and 1, the latter representing high confidence and good signal-to-noise ratio (SNR).

**Liver Biopsy and Histopathology Scoring**

*Biopsy:* Right-lobe percutaneous biopsies were obtained using 16- or 18-gauge needles for clinical care or as part of the parent research studies, fixed in formalin, embedded in paraffin, and stained with hematoxylin-eosin and Masson trichrome. No additional biopsy was obtained for this retrospective analysis. At Center 1 where subjects were enrolled, it is standard practice to acquire at least one 2-cm biopsy, as determined by visual inspection.

*Histopathology scoring:* Regardless of why the biopsy was obtained (clinical care or research), one of two experienced hepatopathologists (each > 10 years of experience) reviewed and scored each biopsy specimen for research using the NASH Clinical Research Network histologic system [[6](#_ENREF_6)]. Fibrosis was scored from 0 to 4, steatosis from 0 to 3, lobular inflammation from 0 to 3, and hepatocellular ballooning from 0 to 2.

**Statistical Analysis**

*Confounders of reproducibility and accuracy:* The potential confounding effects on reproducibility by the following factors were explored: sex, race, BMI, MRI-PDFF, R2*, and the minimum number of pixels in the cumulative ROI by any of the three MRE analysis methods.

This was done using Bayesian Information Criterion (BIC)-based stepwise linear regression models of individual CV for stiffness across the three MRE analysis methods. A similar analysis was used to evaluate the effect of confounders on accuracy: stepwise logistic regression was used to model correct vs. incorrect classification (using histologic classification as reference) by each of the three MRE analysis methods; in the accuracy analysis, the ROI size by each corresponding method was considered a potential confounder rather than the minimum ROI size by any method. Potential confounding factors were as above, as well as the MRE-biopsy time interval.

**Results of Secondary Analyses**

*Confounders of reproducibility:* The BIC-based stepwise regression revealed no confounders for reproducibility between the three analysis methods.

*Confounders of accuracy:* For classifying advanced fibrosis, higher BMI was associated with classification errors by each of the three analysis methods (*P* ranging from 0.015 to 0.02). For classifying any fibrosis, higher PDFF was associated with classification errors by the Center 2 analyst (*P* = 0.016) and automated analysis (*P* = 0.018). No confounders were associated with classification errors by the Center 1 analyst. None of the other covariates examined (age, sex, race, R2*, number of pixels, and time interval between biopsy and MRE examination) were associated with classification errors.

**References**

1 Hamilton G, Yokoo T, Bydder M et al (2011) In vivo characterization of the liver fat (1) H MR spectrum. NMR Biomed 24:784-790

2 Yin M, Talwalkar JA, Glaser KJ et al (2007) Assessment of hepatic fibrosis with magnetic resonance elastography. Clin Gastroenterol Hepatol 5:1207-1213 e1202

3 Chen J, Talwalkar JA, Yin M, Glaser KJ, Sanderson SO, Ehman RL (2011) Early detection of nonalcoholic steatohepatitis in patients with nonalcoholic fatty liver disease by using MR elastography. Radiology 259:749-756

4 Yin M, Talwalkar JA, Glaser KJ et al (2011) Dynamic Postprandial Hepatic Stiffness Augmentation Assessed With MR Elastography in Patients With Chronic Liver Disease. AJR Am J Roentgenol 197:64-70

5 Westin CF, Wigstrom L, Loock T, Sjoqvist L, Kikinis R, Knutsson H (2001) Three-dimensional adaptive filtering in magnetic resonance angiography. J Magn Reson Imaging 14:63-71

6 Kleiner DE, Brunt EM, Van Natta M et al (2005) Design and validation of a histological scoring system for nonalcoholic fatty liver disease. Hepatology 41:1313-1321

**Supplemental Table 1:** Cross-validated Classification Accuracy Parameters of 2D MRE for Staging Liver Fibrosis

| Fibrosis stage classification | MRE  stiffness  cutoff | Sensitivity (%) | Specificity (%) | Accuracy (%) | PPV (%) | NPV (%) |  |
| --- | --- | --- | --- | --- | --- | --- | --- |
| **Center 1 Analyst** | | | | | | | |
| 0 (*n* = 56) vs ≥ 1 (*n* = 35) | 2.99 kPa | 68 (30/44)  [52, 81] | 89 (42/47)  [77, 97] | 79 (72/91)  [69, 87] | 86 (30/35)  [70, 95] | 75 (42/56)  [62, 86] |  |
| ≤ 2 (*n* = 74) vs ≥ 3 (*n* = 17) | 3.60 kPa | 87 (13/15)  [60, 98] | 95 (72/76)  [87, 99] | 93 (85/91)  [86, 98] | 77 (13/17)  [50, 93] | 97 (72/74)  [91, 100] |  |
| **Center 2 Analyst** | | | | | | | |
| 0 (*n* = 58) vs ≥ 1 (*n* = 33) | 2.98 kPa | 64 (28/44)  [48, 78] | 89 (42/47)  [77, 97] | 77 (70/91)  [67, 85] | 85 (28/33)  [68, 95] | 72 (42/58)  [59, 83] |  |
| ≤ 2 (*n* = 74) vs ≥ 3 (*n* = 17) | 3.65 kPa | 87 (13/15)  [60, 98] | 95 (72/76)  [87, 99] | 93 (85/91)  [86, 98] | 77 (13/17)  [50, 93] | 97 (72/74)  [91, 100] |  |
| **Automated Analysis** | | | | | | | |
| 0 (*n* = 64) vs ≥ 1 (*n* = 27) | 3.29 kPa | 50 (22/44)  [35, 65] | 89 (42/47)  [77, 97] | 70 (64/91)  [60, 80] | 82 (22/47)  [62, 94] | 66 (42/64)  [53, 77] |  |
| ≤ 2 (*n* = 73) vs ≥ 3 (*n* = 18) | 3.65 kPa | 87 (13/15)  [60, 98] | 93 (71/76)  [60, 98] | 92 (84/91)  [85, 97] | 72 (13/18)  [60, 98] | 97 (71/73)  [91, 100] |  |

*n* = number of subjects in each dichotomized fibrosis stage. Data in parentheses are raw data, and data in brackets are 95% confidence intervals.

*ROC* = receiver operating characteristic. *PPV* = positive predictive value. *NPV* = negative predictive value. *MRE* = magnetic resonance elastography.

**Supplemental Table 2.** Pairwise comparisons of performance parameters for classifying presence of significant fibrosis and advanced fibrosis. *P-*values of 0.0042 (0.05/12) or less can be considered significant at the family-wise 0.05 level after the Bonferroni adjustment.

| Performance metric | Comparison | Fibrosis stage | Estimate | *P*-value |
| --- | --- | --- | --- | --- |
| AUC | Center 1 analyst vs. automated analysis | 0 vs ≥ 1 | 0.001 (-0.045, 0.052) | 0.95 |
|  |  | ≤ 2 vs ≥ 3 | -0.008 (-0.049, 0.016) | 0.30 |
|  | Center 1 analyst vs. Center 2 analyst | 0 vs ≥ 1 | -0.013 (-0.091, 0.050) | 0.51 |
|  |  | ≤ 2 vs ≥ 3 | -0.001 (-0.033, 0.018) | 0.71 |
|  | Center 2 analyst vs. automated analysis | 0 vs ≥ 1 | -0.014 (-0.091, 0.053) | 0.58 |
|  |  | ≤ 2 vs ≥ 3 | 0.007 (-0.017, 0.050) | 0.41 |
| Sensitivity | Center 1 analyst vs. automated analysis | 0 vs ≥ 1 | 0.182 (0.024, 0.395) | **0.001** |
|  |  | ≤ 2 vs ≥ 3 | 0.000 (-0.385, 0.000) | 0.95 |
|  | Center 1 analyst vs. Center 2 analyst | 0 vs ≥ 1 | 0.023 (-0.152, 0.183) | 0.24 |
|  |  | ≤ 2 vs ≥ 3 | 0.000 (-0.385, 0.000) | 0.95 |
|  | Center 2 analyst vs. automated analysis | 0 vs ≥ 1 | -0.433 (-0.739, -0.181) | **0.001** |
|  |  | ≤ 2 vs ≥ 3 | 0.000 (-0.467, 0.000) | 0.95 |
| Specificity | Center 1 analyst vs. automated analysis | 0 vs ≥ 1 | -0.021 (-0.100, 0.053) | 0.200 |
|  |  | ≤ 2 vs ≥ 3 | 0.013 (-0.069, 0.065) | 0.44 |
|  | Center 1 analyst vs. Center 2 analyst | 0 vs ≥ 1 | -0.021 (-0.100, 0.059) | 0.190 |
|  |  | ≤ 2 vs ≥ 3 | 0.000 (-0.096, 0.043) | 0.36 |
|  | Center 2 analyst vs. automated analysis | 0 vs ≥ 1 | 0.010 (-0.058, 0.099) | 0.36 |
|  |  | ≤ 2 vs ≥ 3 | -0.013 (-0.068, 0.063) | 0.40 |
| Accuracy | Center 1 analyst vs. automated analysis | 0 vs ≥ 1 | 0.077 (0.000, 0.198) | **0.002** |
|  |  | ≤ 2 vs ≥ 3 | 0.011 (-0.066, 0.044) | 0.62 |
|  | Center 1 analyst vs. Center 2 analyst | 0 vs ≥ 1 | 0.000 (-0.121, 0.044) | 0.450 |
|  |  | ≤ 2 vs ≥ 3 | 0.000 (-0.077, 0.037) | 0.40 |
|  | Center 2 analyst vs. automated analysis | 0 vs ≥ 1 | -0.077 (-0.187, 0.000) | **0.004** |
|  |  | ≤ 2 vs ≥ 3 | -0.011 (-0.062, 0.044) | 0.47 |
